# Supplementary material for: Double Blast Wave Primary Effect on Synaptic, Glymphatic, Myelin, Neuronal and Neurovascular Markers
Source: Brain Sci. 2023 Feb 8;13(2):286. doi: 10.3390/brainsci13020286 (PMC9954059; doi:10.3390/brainsci13020286)
Supplement: Supplementary file 1 [file brainsci-13-00286-s001.zip › brainsci-2184669-supplementary.pdf]

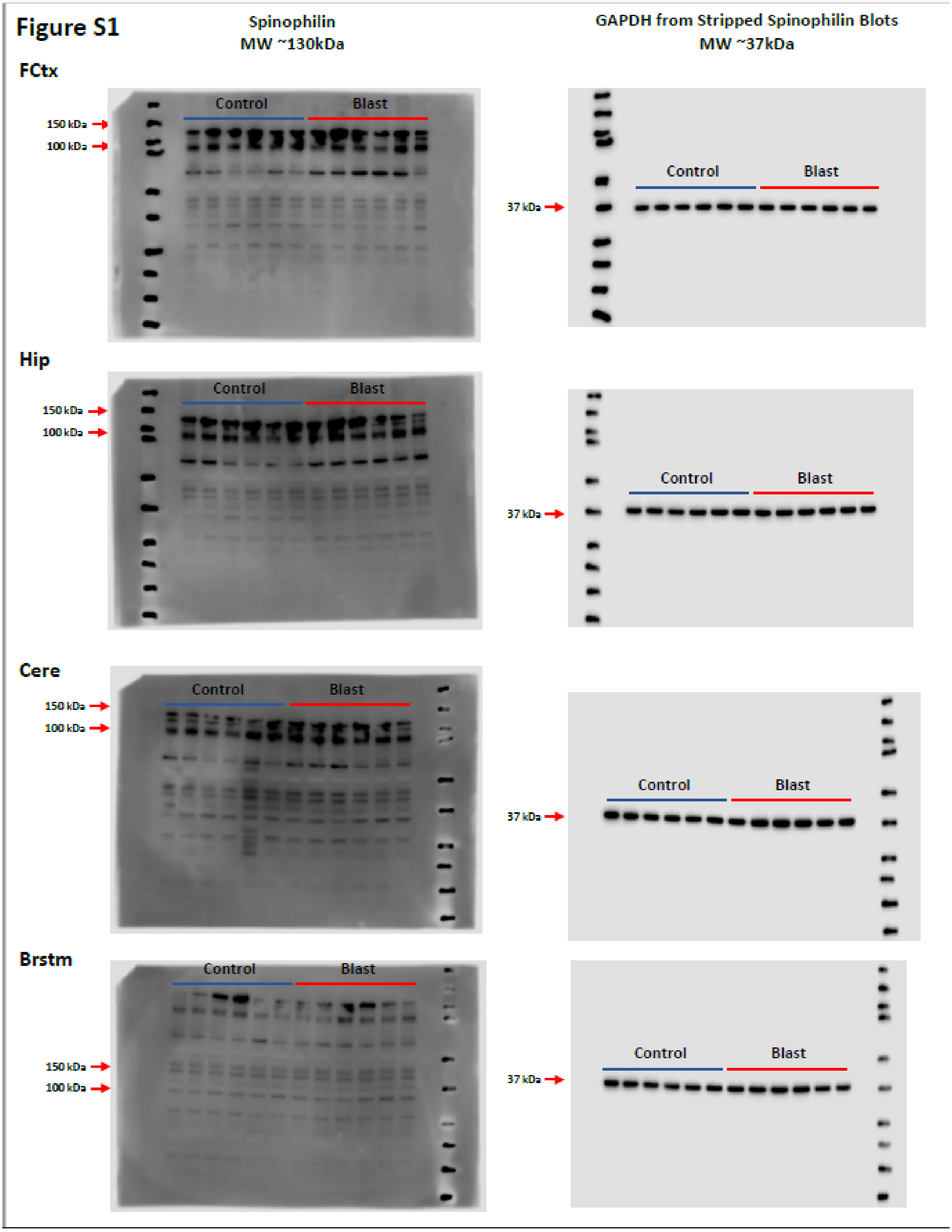

Figure S1. Representative full length western blots for Spinophilin and associated GAPDH.

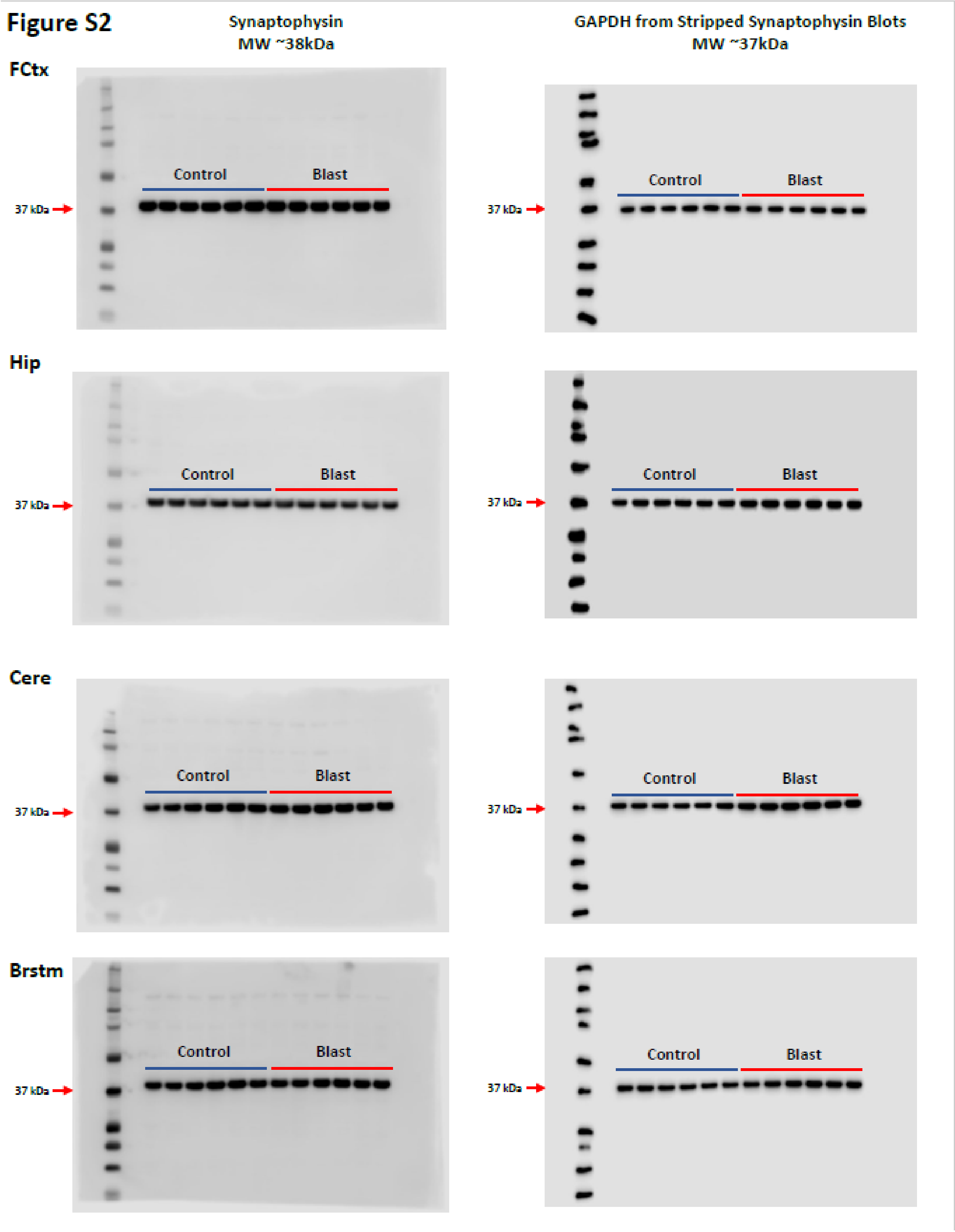

Figure S2. Representative full length western blots for Synaptophysin and associated GAPDH.

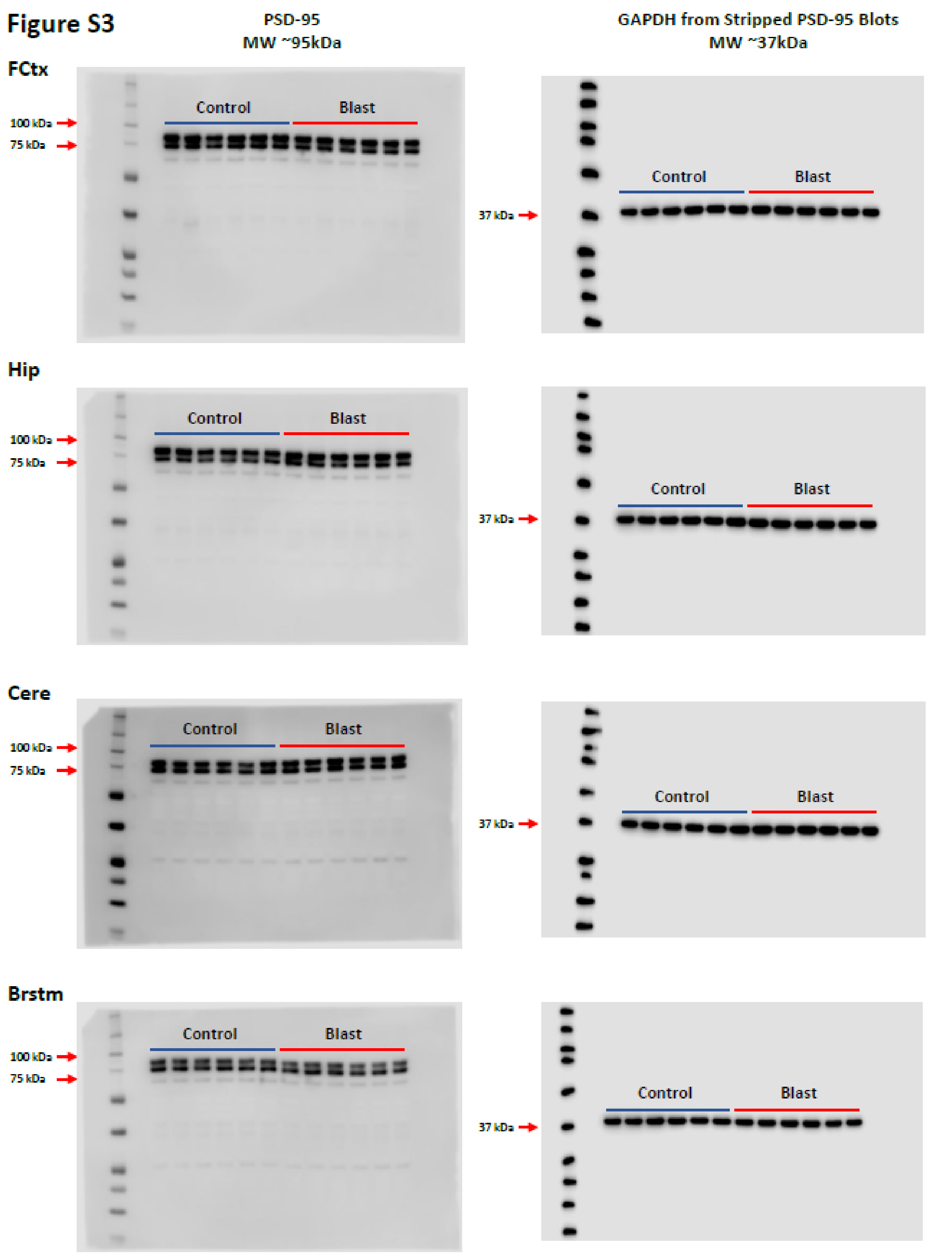

Figure S3. Representative full length western blots for PSD-95 and associated GAPDH.

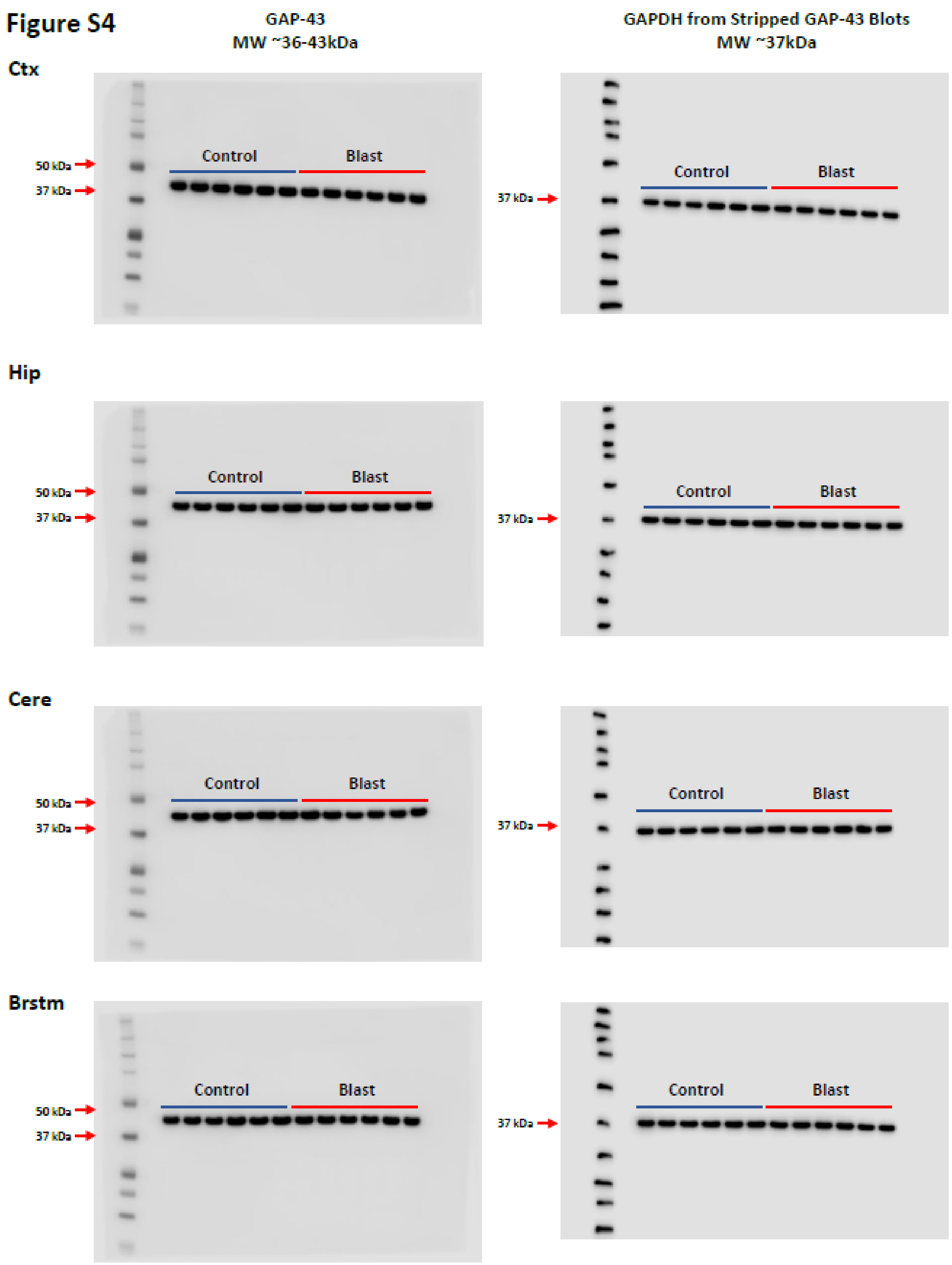

Figure S4. Representative full length western blots for GAP-43 and associated GAPDH.

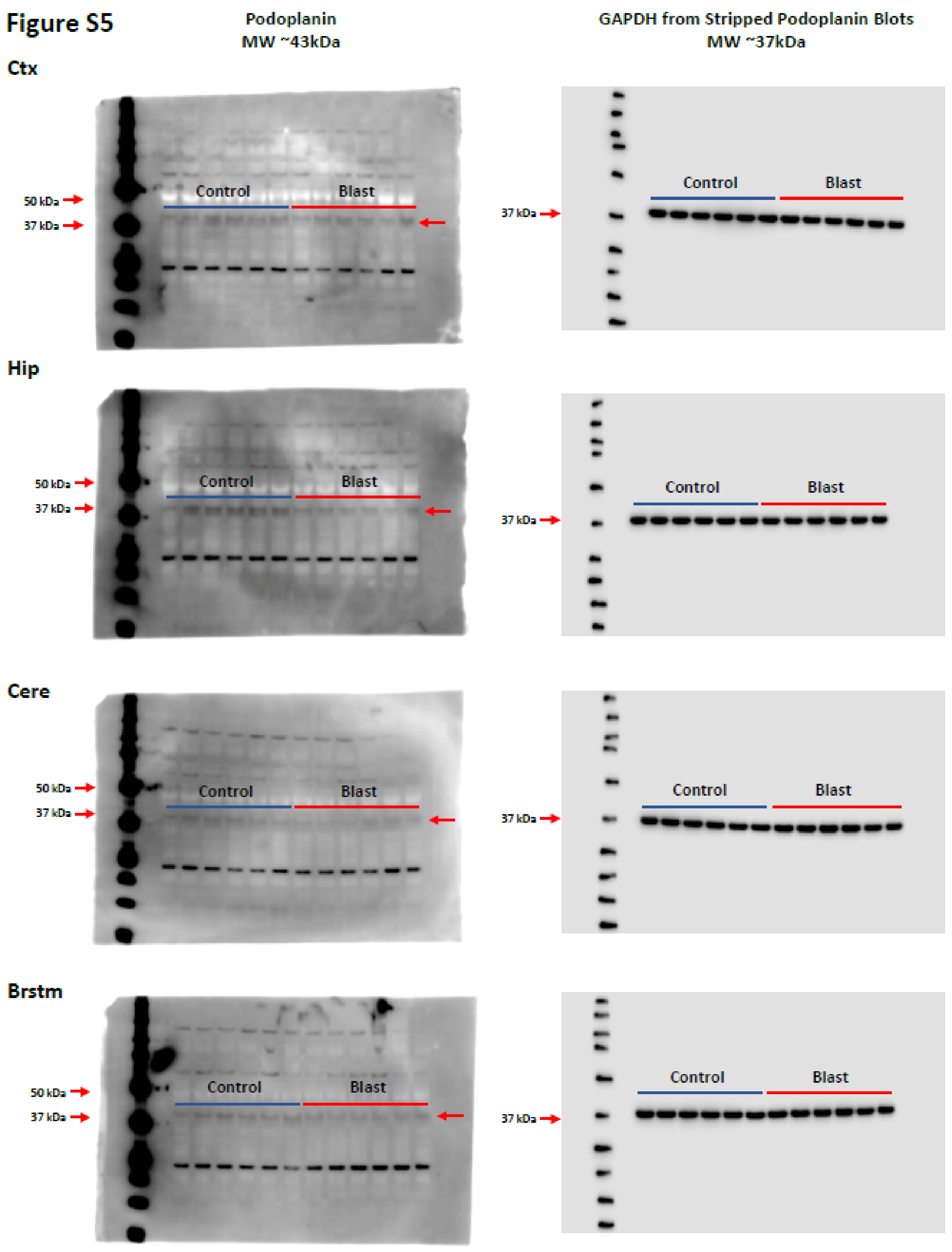

Figure S5. Representative full length western blots for Podoplanin and associated GAPDH.

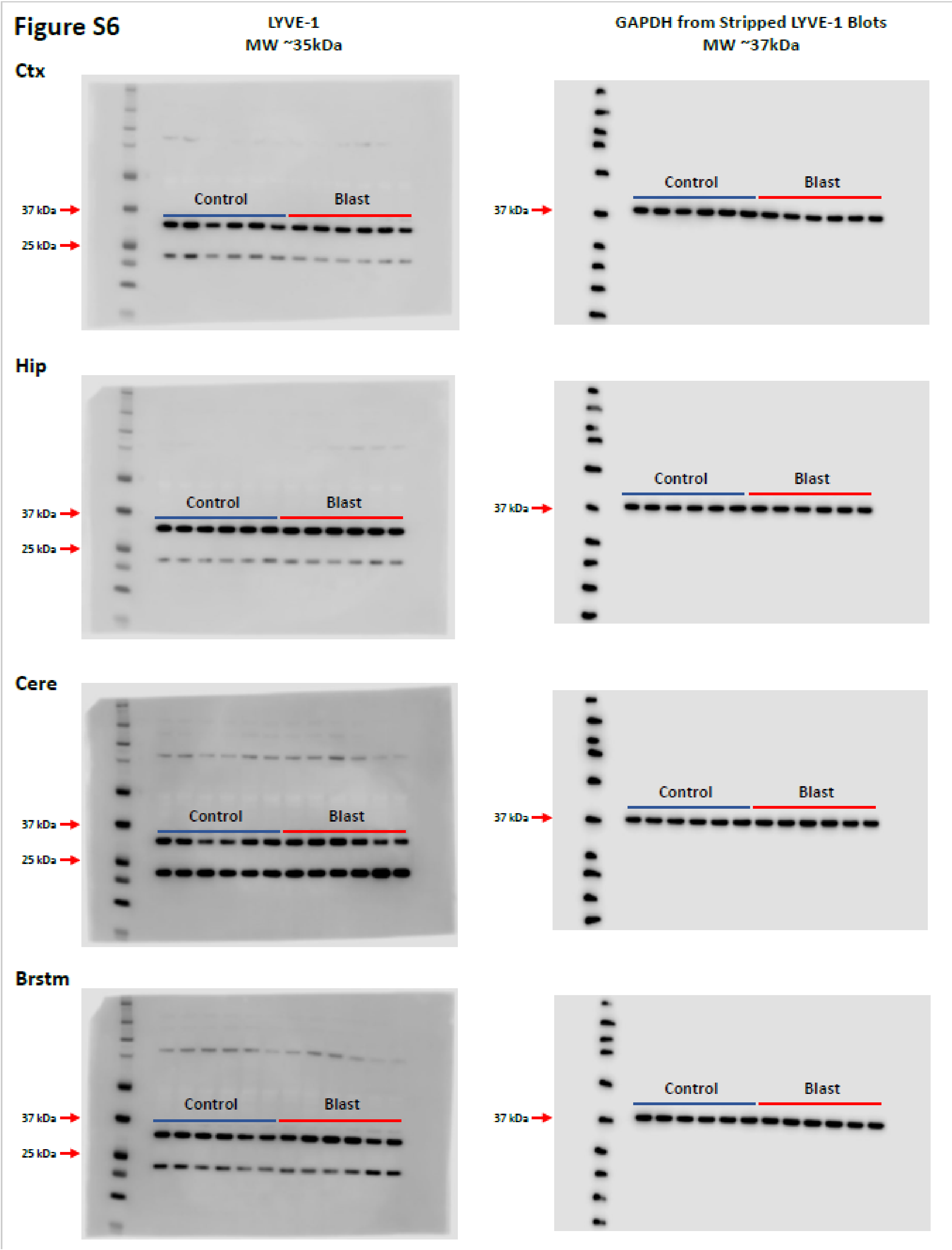

Figure S6. Representative full length western blots for LYVE1 and associated GAPDH.

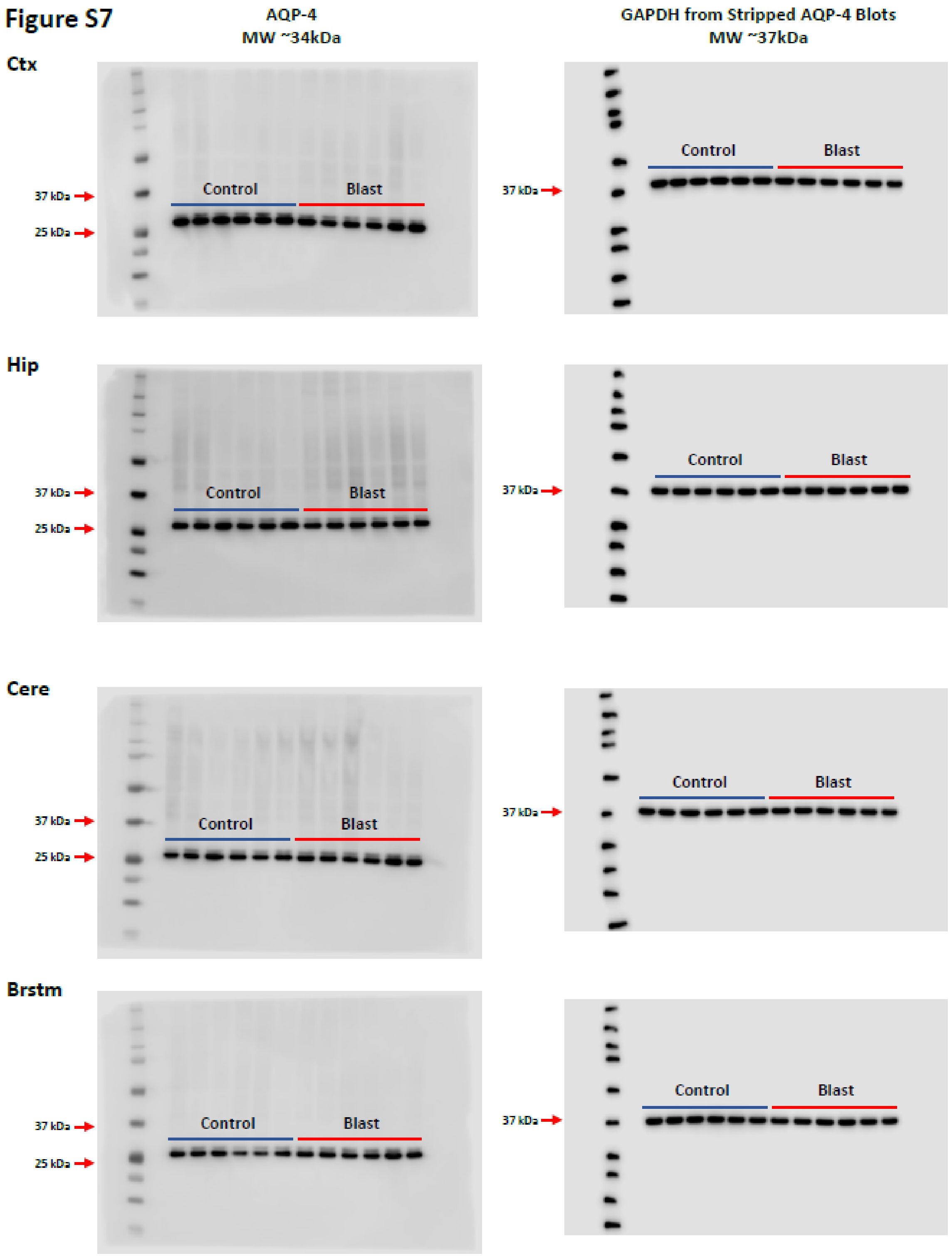

Figure S7. Representative full length western blots for AQP4 and associated GAPDH.

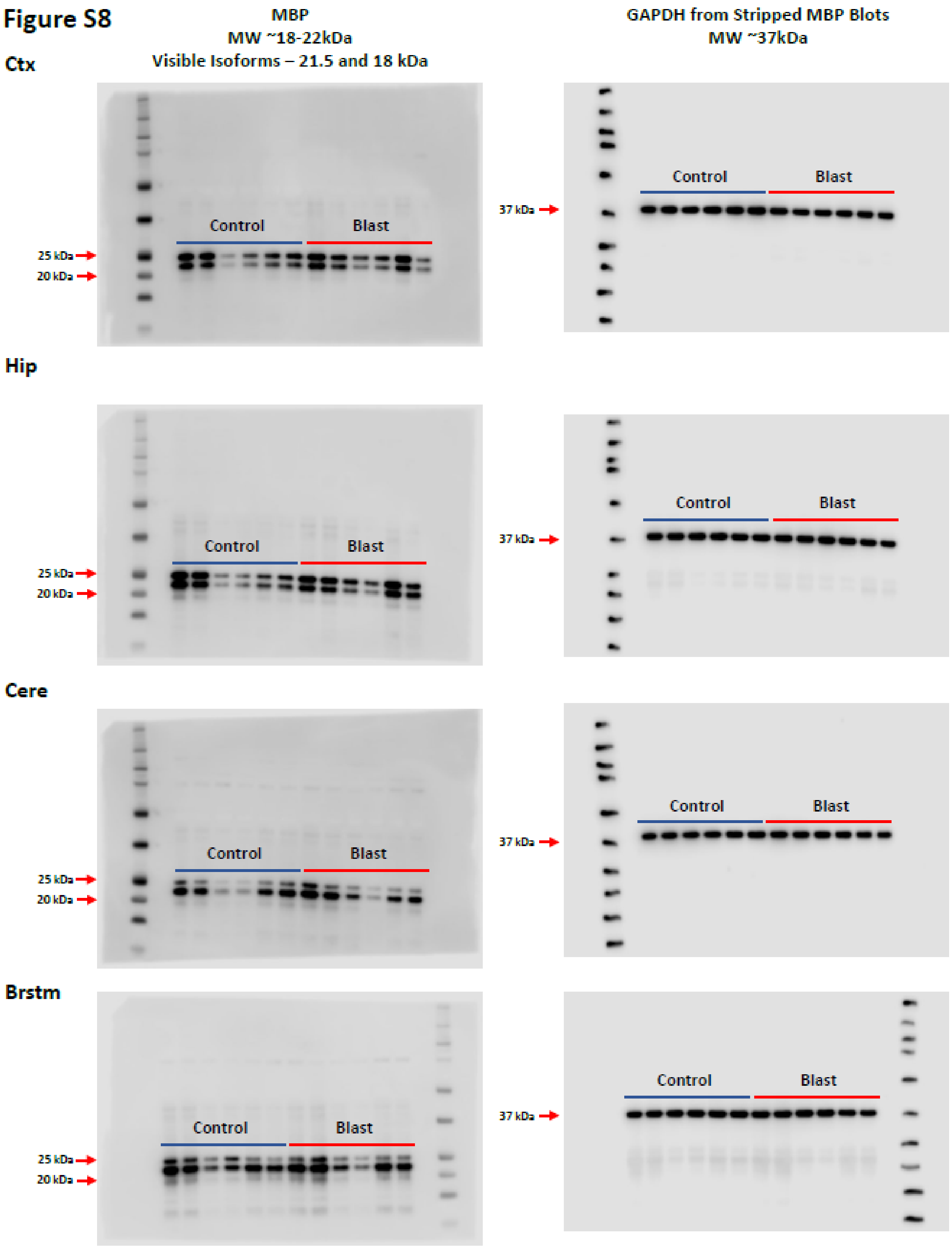

Figure S8. Representative full length western blots for MBP and associated GAPDH.

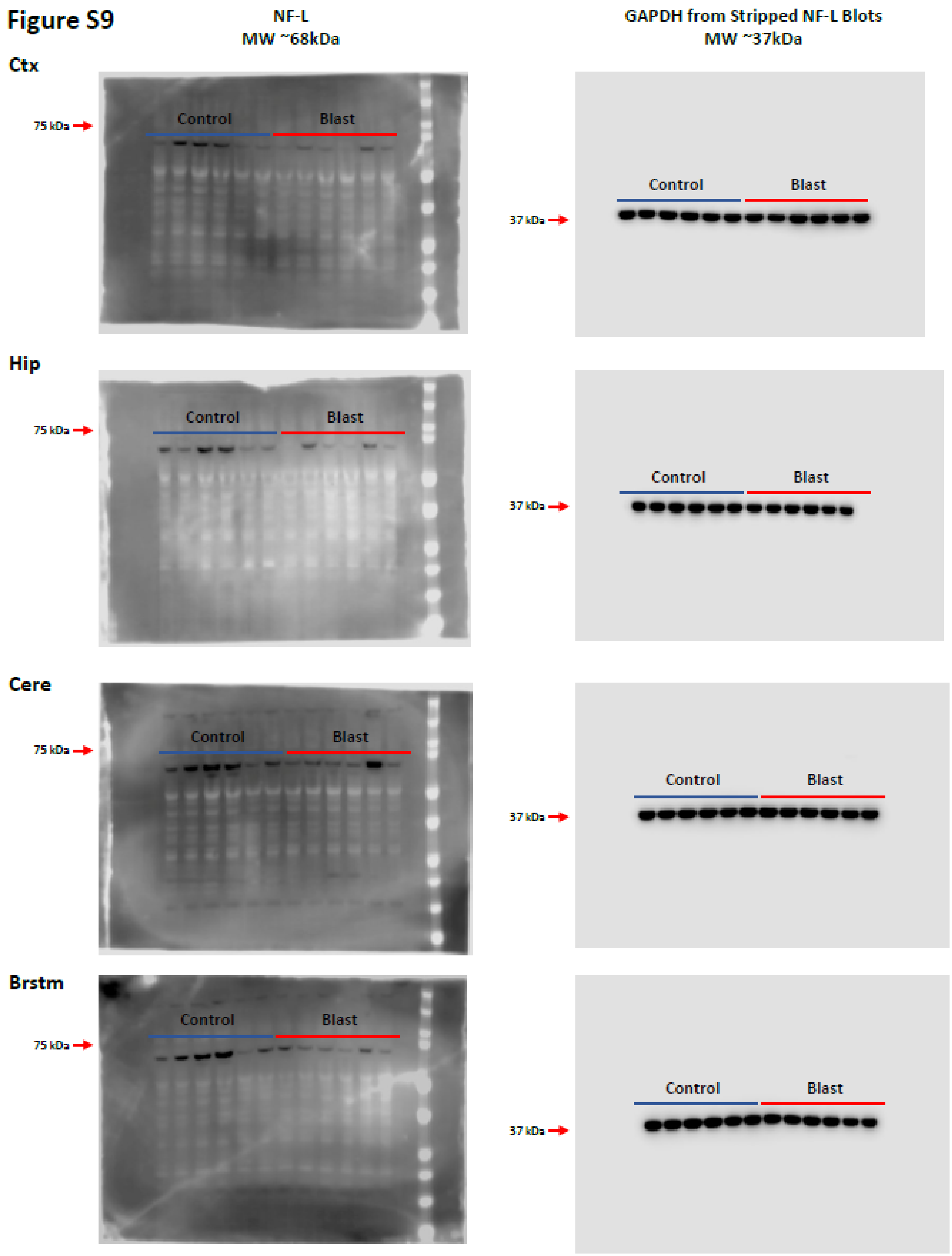

Figure S9. Representative full length western blots for NF-L and associated GAPDH.

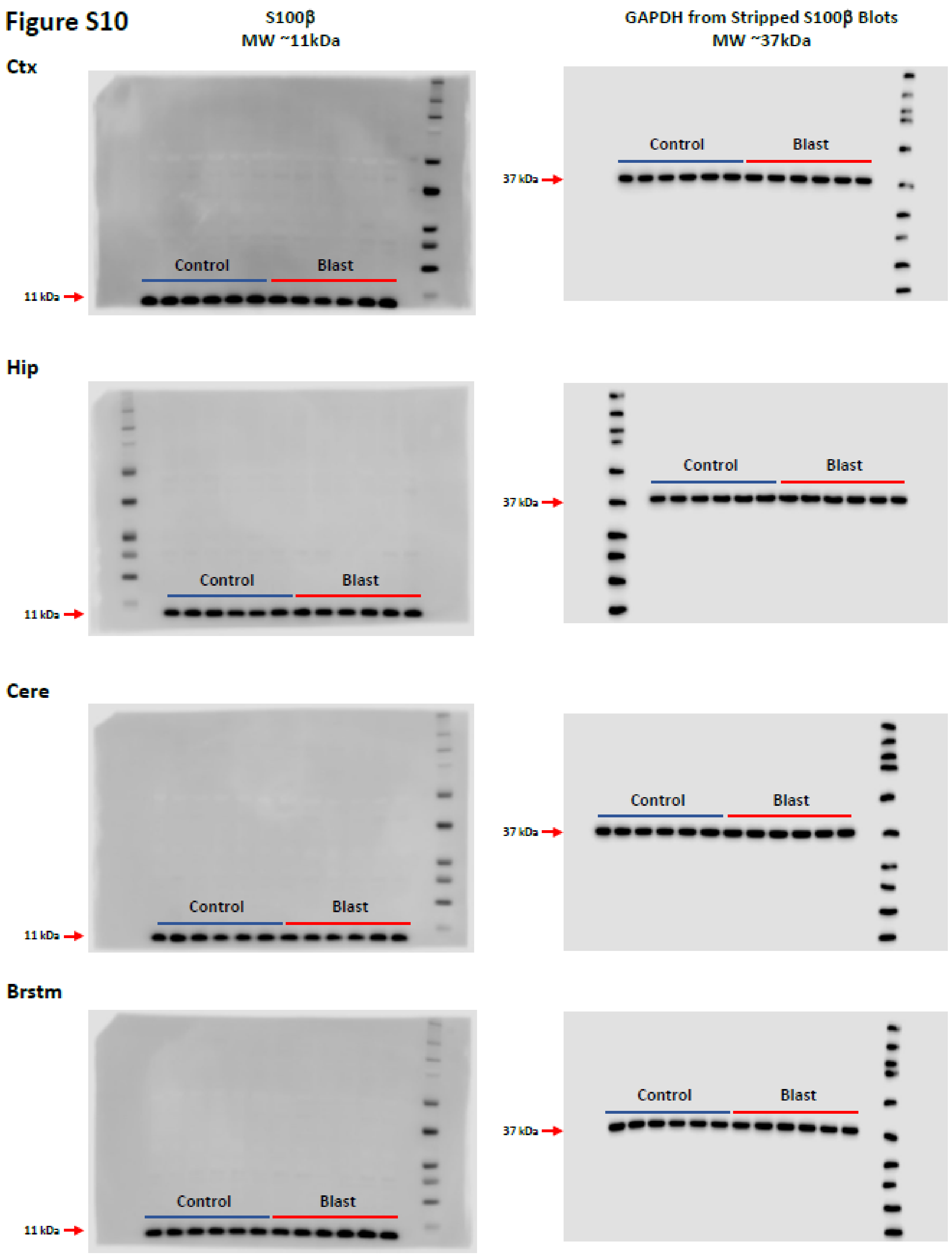

Figure S10. Representative full length western blots for S100β and associated GAPDH.

Figure S11

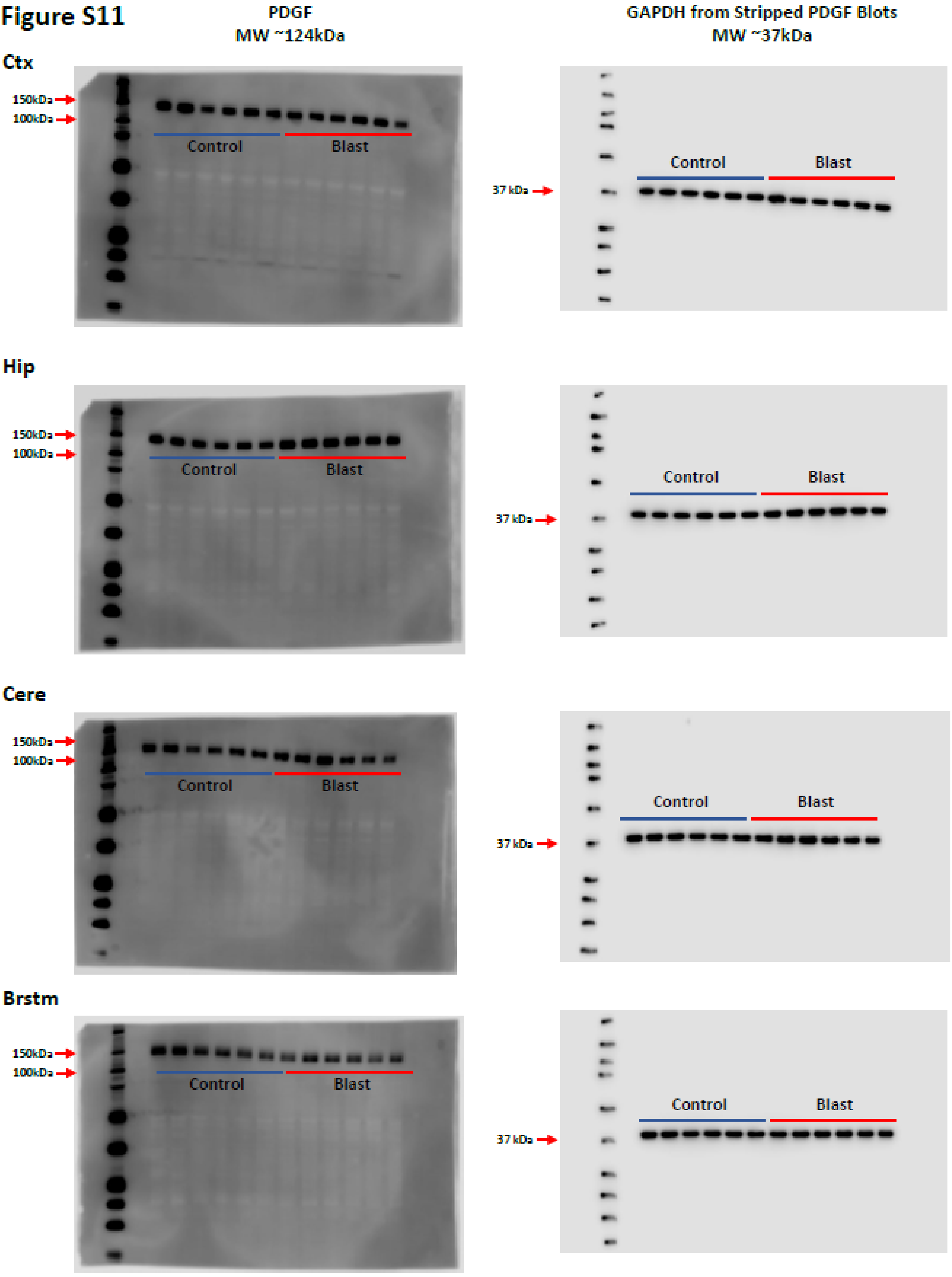

Figure S11. Representative full length western blots for PDGF and associated GAPDH.

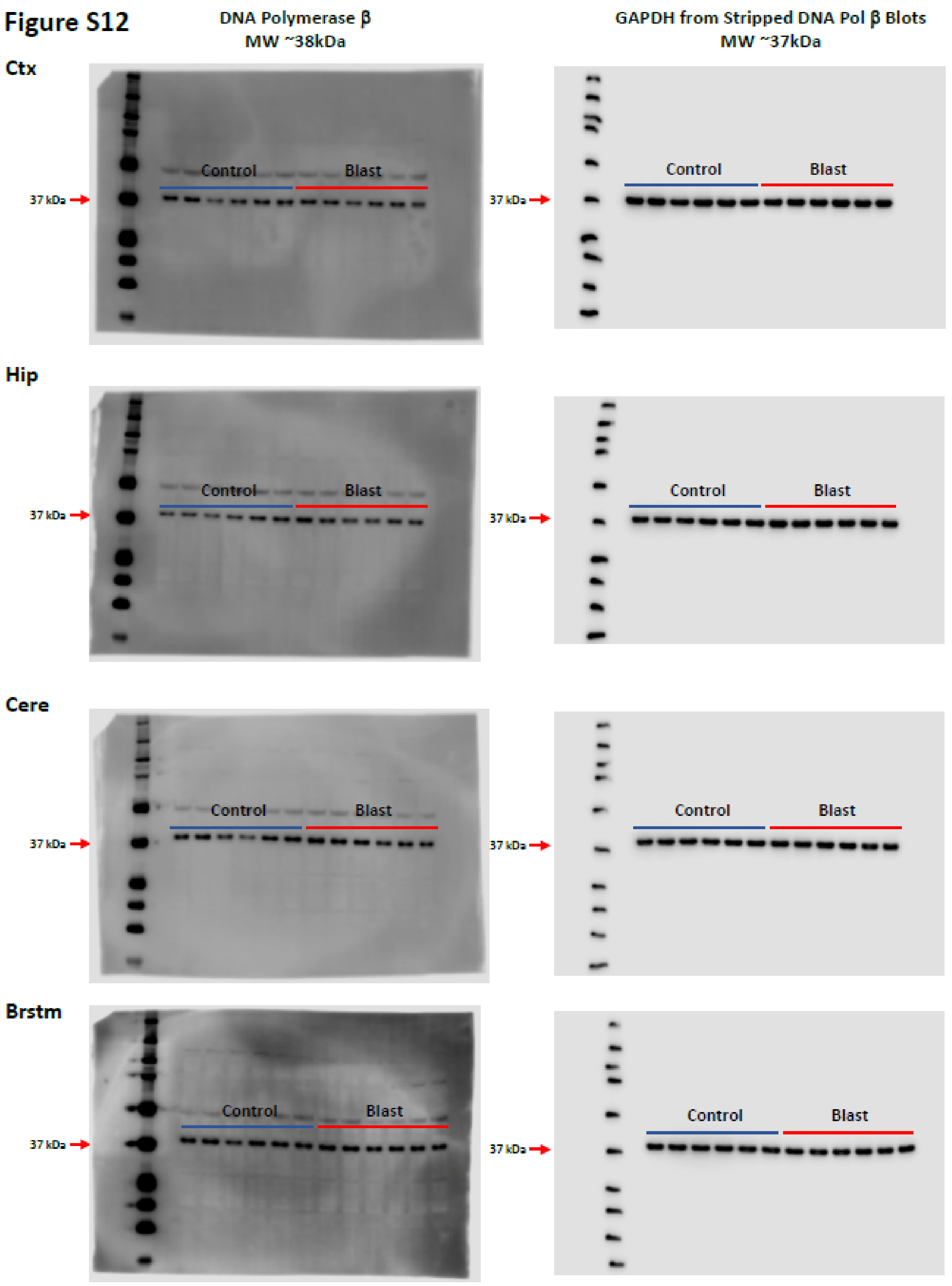

Figure S12. Representative full length western blots for DNA Polymerase β and associated GAPDH.

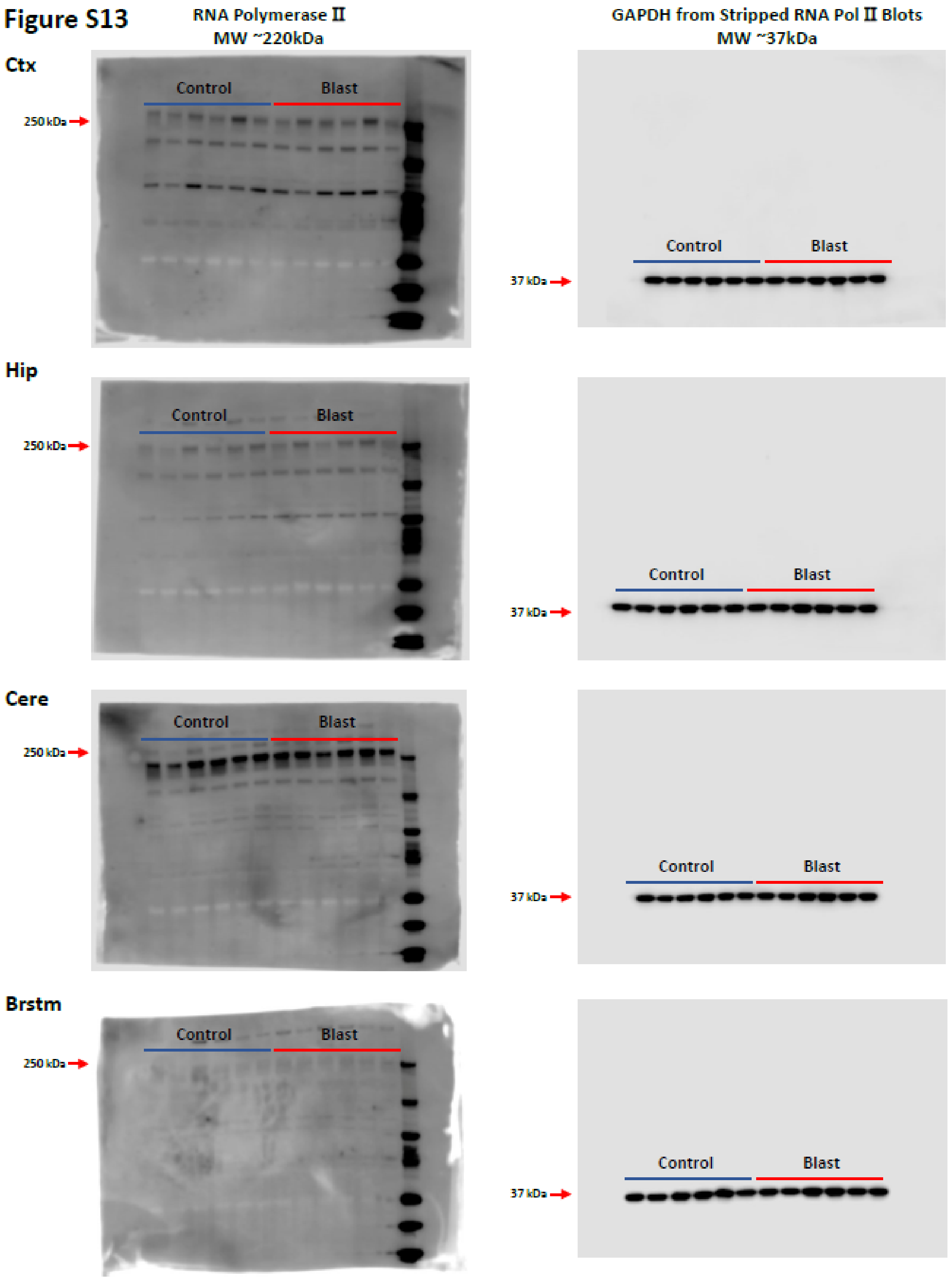

Figure S13. Representative full length western blots for RNA Polymerase II and associated GAPDH.
